# Genomic Features, Functional Complementarity, and the Potential for Constructing Synthetic Consortia from Dominant Petroleum-Degrading Bacteria

**DOI:** 10.3390/biology15161401

**Published:** 2026-08-15

**Authors:** Fei Zhang, Xiuyue Xiao, Shuhua Zhu, Runsheng Yin, Shuhan Zhang, Cheng Peng, Xiaopeng Guo, Yonggang Wang, Dong Lu, Zheng Cao

**Affiliations:** 1 School of Life Science and Engineering, Lanzhou University of Technology, Lanzhou 730050, China; 2 Ministry of Education Gansu Advanced Research Institute, Lanzhou 730000, China; 3 Institute of Modern Physics, Chinese Academy of Sciences, Lanzhou 730000, China; 4 School of Automation and Electrical Engineering, Lanzhou University of Technology, Lanzhou 730050, China

**Keywords:** hydrocarbon-degrading isolates, designed microbial assemblages, comparative genome analysis, catabolic enzyme complementarity, polycyclic aromatic hydrocarbons (PAHs)

## Abstract

Microbial remediation serves as a cost-effective and eco-friendly tactic for petroleum-contaminated sites. In real-world practices, petroleum-degrading microbial consortia display prominent superiority over single strains. To tap into their intrinsic synergistic degradation capacity, it is essential to dissect microbial commonalities, individual traits and functional complementarity for complicated bioremediation scenarios. This study applied bibliometric approaches to collect 15 well-documented representative petroleum-hydrocarbon-degrading strains, and summarized research progress via comparative genomic analysis. *Pseudomonas aeruginosa*, *Bacillus subtilis* and *Rhodococcus erythropolis* are the most frequently reported degraders with remarkable petroleum removal performance. Moreover, co-occurrence network analysis identifies *Pseudomonas*, *Bacillus*, *Acinetobacter* and *Rhodococcus* as core co-existing genera sustaining natural pollutant-degrading communities. Genomic evidence indicates these strains harbour abundant functional elements encoding diverse oxygenases, alcohol dehydrogenases, cytochrome P450 monooxygenases and key LuxR-, AraC- and GntR-family regulatory factors. *P. aeruginosa* has the highest copy numbers of catabolic enzyme genes, consistent with its outstanding degradation phenotype. Degraders from different genera exhibit high genetic heterogeneity, with accessory gene clusters significantly enriched in hydrocarbon degradation pathways. Six strains including *P. aeruginosa* and *R. erythropolis* assemble a complete gene cascade targeting recalcitrant polycyclic aromatic hydrocarbons. This work provides reliable genomic support for rational strain screening and synthetic-consortium optimization, facilitating knowledge-driven strategies for efficient petroleum bioremediation.

## Simple Summary

Petroleum pollution is a serious environmental problem because crude oil contains many toxic compounds that are difficult to break down naturally. Using bacteria to clean up oil spills is an eco-friendly approach, but no single bacterial strain can degrade all components of crude oil by itself. Scientists are therefore designing synthetic bacterial consortia—mixed groups of different bacterial species that work together. In this study, we analyzed the complete genomes of 15 representative oil-degrading bacteria to understand their genetic differences. We found that these strains share very few common genes, and the key genes responsible for oil degradation are mostly unique to individual strains. This diversity means different bacteria have different degradation skills that can complement each other. For instance, some strains are better at breaking down polycyclic aromatic hydrocarbons, while others excel at degrading alkanes. By understanding these genomic differences, we can rationally select and combine specific strains to build highly efficient bacterial communities for petroleum cleanup. Our work provides a theoretical foundation for designing tailored bioremediation strategies that are more effective than using single strains alone.

## 1. Introduction

Petroleum pollutants are characterized by high toxicity and structural stability, rendering them recalcitrant to natural attenuation. Their persistence not only undermines biodiversity but also poses substantial threats to human health. Owing to its cost effectiveness and eco-compatibility, microbial remediation has emerged as a cornerstone technology for rehabilitating petroleum-contaminated sites. Genera such as *Pseudomonas*, *Bacillus, Rhodococcus*, *Alcanivorax*, *Marinobacter*, *Acinetobacter*, and *Microbulbifer* exhibit exceptional hydrocarbon-metabolizing capabilities and environmental robustness, establishing them as pivotal germplasm resources for bioremediation [1]. The proliferation of high-throughput sequencing has further elucidated the genetic underpinnings of these degraders; for instance, the complete genome sequence of *Pseudomonas aeruginosa* N002 has systematically revealed the molecular basis of its adaptation to crude oil environments [2].

Crude oil comprises abundant hydrophobic polycyclic aromatic hydrocarbons (PAHs) and long-chain alkanes. Single isolates often suffer from limited substrate spectra, precluding the complete mineralization of such complex matrices. Consequently, constructing synthetic consortia based on multi-strain metabolic synergy and enzymatic cooperation has become a central research focus. Empirical evidence indicates that multi-strain consortia significantly outperform monocultures in crude oil degradation. Moreover, the exogenous addition of *Pseudomonas* spp. or biosurfactants (e.g., Alpha Olefin Sulfonate) can directionally enrich key degraders such as *Alcanivorax* and *Sphingobacter*, thereby optimizing community architecture and enhancing remediation efficacy [3,4].

Nevertheless, metabolic intermediates generated by specific strains can exert stimulatory or inhibitory effects on syntrophic partners; thus, unguided random assemblages frequently fail to yield satisfactory outcomes [5,6]. Current strategies for consortium construction are dichotomized into top-down and bottom-up approaches. The former relies on environmental enrichment to screen native microbial communities—a method that is facile yet lacks precision. Conversely, the bottom-up approach entails the targeted assembly of strains informed by genomic data and metabolic network analysis. By leveraging interspecies metabolic complementarity, this approach represents the vanguard of precision bioremediation. Both mathematical modeling and empirical assays confirm that consortia engineered based on distinct strain functionalities exhibit far superior degradation capacities for heavy oil and crude oil compared to single strains [7,8,9].

Regrettably, existing pan-genomic and comparative genomic studies predominantly focus on single species, lacking systematic, cross-generic comparisons among petroleum-degrading bacteria. This knowledge gap impedes the comprehensive exploration of functional complementarity patterns across taxa. To address this limitation, the present study conducted a bibliometric analysis utilizing the Web of Science Core Collection to identify 15 representative hydrocarbon-degrading strains distinguished by high-quality complete genomes and extensive scholarly coverage. Publicly available genomic datasets for these strains were retrieved. Innovatively extrapolating pan-genome analysis—traditionally confined to single species—to encompass multiple genera, this study employed comparative genomics as the primary methodology to systematically dissect shared attributes, strain-specific divergences, and metabolic complementary potential. By integrating phenotypic and genotypic perspectives, we delineated variations in substrate degradation profiles across the 15 strains. These findings provide genomic-level theoretical support for the rational selection of species and the optimization of their proportions in the design of synthetic consortia for petroleum bioremediation.

## 2. Materials and Methods

### 2.1. Bibliometric Screening and Genomic Dataset Curation

Relevant literature was retrieved from the Web of Science (WOS) Core Collection using the following search string: TS = ((“microorganism” OR “bacteria” OR “microbiome” OR “bacterium”) AND (“petroleum hydrocarbon” OR “oil pollution” OR “crude oil”) AND (“biodegradation” OR “bioremediation” OR “degradation”)). Publications from 2010 to 2025 were included, yielding 4749 records. Bibliometric parameters, including citation frequency and research hotspots, were analyzed using HistCite Pro 2.1, R 4.4.2, and Microsoft Excel 2019 softwares. To quantify the academic impact of specific strains, a citation score was calculated for each key publication using the formula: Score = Citation Frequency/(Number of Records Published Since the Target Paper).

Candidate strains were preliminarily screened based on four objective criteria: (i) high reporting frequency in the WOS database; (ii) well-documented hydrocarbon-degrading capabilities; (iii) degradation phenotypes supported by highly cited literature; and (iv) broad taxonomic coverage ensuring representative species from diverse genera. A secondary screening refined this list by evaluating three quantitative metrics: reported degradation efficiency, public availability of genomic data, and assembly quality (chromosome-level or above). Optimal assemblies—prioritizing completeness, sequence length, and public accessibility—were downloaded from the NCBI database. Ultimately, 15 strains possessing both high-quality, chromosome-level genomes and literature-validated degradation phenotypes were selected. These included *Pseudomonas aeruginosa* RW109, *Bacillus subtilis* PRO122, *Rhodococcus erythropolis* X5, *Acinetobacter baumannii* AC1633, *Alcaligenes faecalis* NY11312, *Enterobacter hormaechei* MRSN791417, *Stenotrophomonas maltophilia* SM866, *Lysinibacillus fusiformis* NEB1292, *Serratia marcescens* CM2015_854, *Aeribacillus pallidus* KCTC3564, *Aeromonas hydrophila* K533, *Chelatococcus daeguensis* TAD1, *Dietzia lutea* YIM80766, *Exiguobacterium aurantiacum* CX2, and *Kocuria flava* HO-9041. The 15 selected strains, along with their corresponding genome accession numbers, are listed in Table S1.

### 2.2. Genome-Wide Annotation of Hydrocarbon Degradation Pathways

Complete genome sequences (.fna files) of the 15 strains were subjected to systematic functional annotation. A phylogenetic tree was reconstructed using the Neighbor-Joining (NJ) method implemented in MEGA 12 software, with bootstrap values calculated from 1000 replicates. Gene prediction and structural annotation were performed using Prokka (v1.14.6). Functional annotations were integrated from multiple databases, including GO, COG, KEGG (orthology-based), NR, SwissProt, TrEMBL (sequence similarity-based), and Pfam (domain-based), to establish a comprehensive reference gene set specific to hydrocarbon degradation. The genomic landscape of degradation genes—including gene counts, chromosomal distribution density, and proportional representation—was visualized using TBtools 2.056 software and the ggplot2 package in R (v4.4.2).

### 2.3. Comparative Genomics and Pan-Genome Analysis

To elucidate the genetic diversity underlying biodegradation, we extended pan-genome analysis beyond single species to encompass multiple genera. Roary (v3.13.0) was used to construct the gene presence/absence matrix, with result processing and visualization performed via R (v4.4.2) and Python (v3.12.13) scripting. Direct orthology analysis was conducted on GFF files using BLASTP (2.17.0) to delineate the core and accessory genomes. Orthologous gene clusters were defined as gene groups present in 11 or more strains. Genomic collinearity and rearrangement events were visualized using the R packages devtools and RIdeogram, highlighting conserved syntenic blocks across the selected strains.

### 2.4. Co-Occurrence Network Construction Based on Bibliometric Data

To map ecological interactions, 100 highly cited publications regarding petroleum remediation were curated to extract taxonomic compositions of both natural and synthetic consortia. A co-occurrence matrix was constructed in Rstudio (2022.12.0-353). The top 30 dominant taxa, ranked by cumulative co-occurrence frequency, were selected for network visualization. A chord diagram depicting inter-taxonomic associations was generated using the D, providing a visual representation of the strength and directionality of interactions among core degraders.

### 2.5. Assessment of Interspecies Metabolic Complementarity

Potential metabolic complementarity among strains was evaluated through an integrative framework combining bibliometrics, comparative genomics, and network analysis. Initially, bibliometric data identified hotspot strains and inferred phenotypic complementarity. Subsequently, differential gene analysis based on functional annotations revealed genetic disparities in biodegradation potential. The pan-genome partitioning distinguished whether degradation determinants resided in the stable core or the flexible accessory genome. Finally, KEGG pathway enrichment analysis uncovered potential cross-feeding and co-metabolic patterns. This multi-omics approach elucidated the genetic and metabolic basis of interspecies synergy from phenotypic, genotypic, and pathway perspectives.

### 2.6. Statistical Analysis

Structural annotation via Prokka was executed with a default e-value threshold of 10^−6^. Orthologous clustering using Roary employed a BLASTP identity cutoff of 95%. Core genes were strictly defined as those present in ≥11 of the 15 strains. KEGG pathway mapping was performed against the KEGG GENES database based on Prokka-assigned KO identifiers. Statistical significance of pathway enrichment was determined using a hypergeometric test, followed by Benjamini–Hochberg (BH) correction for multiple hypothesis testing. Pathways with a false discovery rate (FDR) < 0.05 were considered statistically significant.

## 3. Results

### 3.1. Evolution of Keywords, Strain Reporting Frequency, and Degradation Characteristics in the Literature

#### 3.1.1. Keyword Evolution Indicates the Significance of Different Strains in Petroleum Hydrocarbon Biodegradation

**Figure 1:**
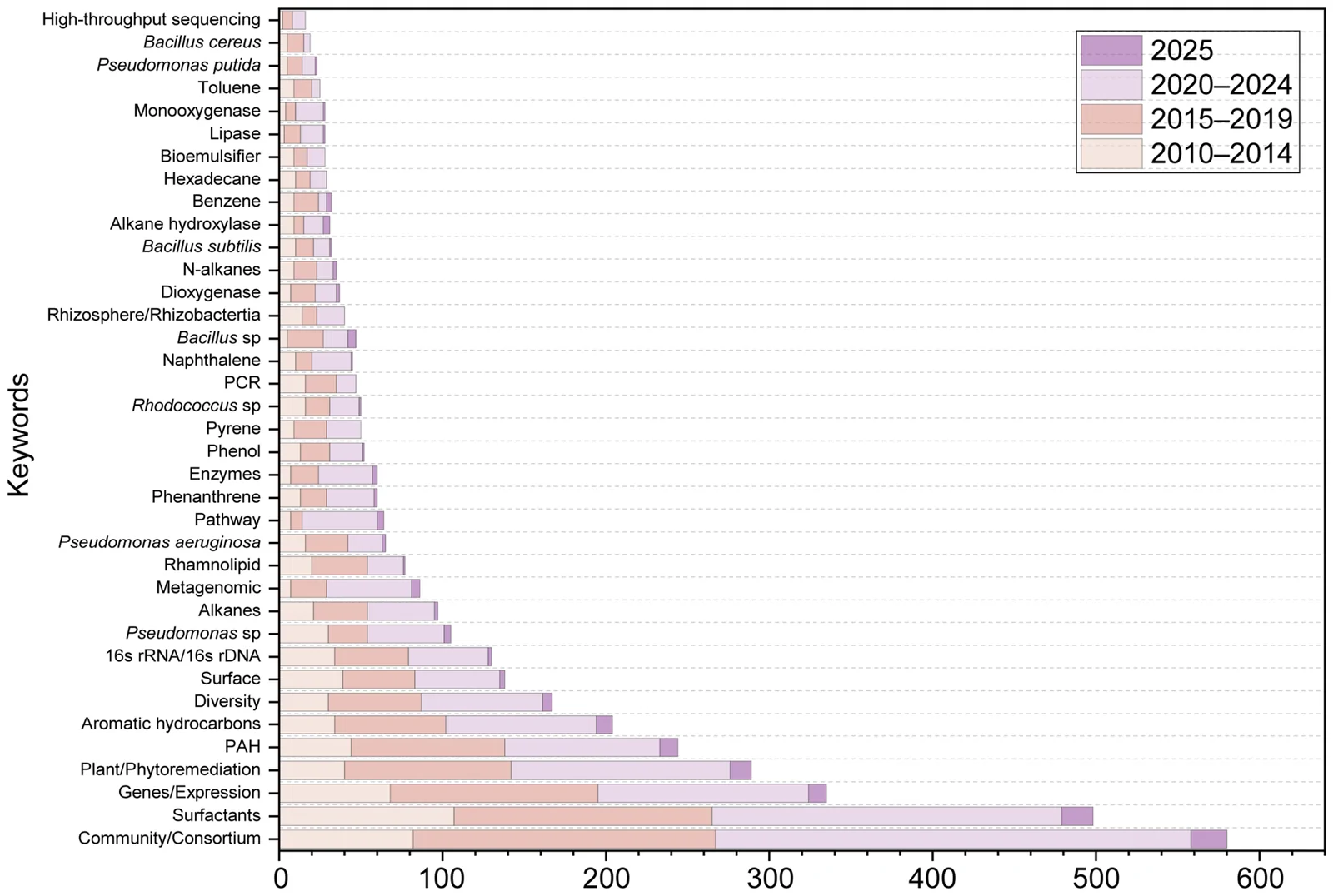
Comparison of main keywords in the literature on microbial degradation of petroleum hydrocarbons over the past 16 years. The quantitative criterion for keyword inclusion was a total frequency greater than 20 within the search period. The literature data were updated as of 31 December 2025. Abbreviations: Polycyclic Aromatic Hydrocarbon (PAH); Polymerase Chain Reaction (PCR).

Bibliometric data, acquired as outlined in Section 2.1, were stratified into four chronological phases (2010–2014, 2015–2019, 2020–2024, and 2025) to map the evolving research landscape of microbial petroleum hydrocarbon degradation. Keywords with a cumulative frequency >20 were subjected to quantitative analysis (Figure 1). The results revealed that “Community/Consortium” consistently exhibited the highest frequency across all periods. While keywords such as “16S rRNA/16S rDNA” and “*Pseudomonas*” dominated the early phase (2010–2014), their prevalence diminished over time concomitant with deepening research. Notably, “*Pseudomonas aeruginosa*” appeared significantly more frequently than any other specific strain. The emergence of terms such as “Plant/Phytoremediation” as well as “Rhizo-sphere/Rhizobacteria” signaled a growing interest in plant–microbe interactions. Furthermore, the prominence of “PAH”—ranked fifth—as well as the appearance of “Phenanthrene” and “Aromatic hydrocarbons”, reflected targeted investigations into recalcitrant petroleum constituents. Finally, the high ranking of “Gene/Expression”, coupled with the emergence of “16S rRNA/16S rDNA”, “High-throughput sequencing”, and non-illustrated terms (e.g., Illumina sequencing, Next-generation sequencing, Annotation, Metagenomic analysis, and Proteomic analysis), evidenced the extensive integration of multi-omics technologies in petroleum remediation research.

#### 3.1.2. Statistics on Microbial Species with Reported Petroleum Hydrocarbon Degradation Capabilities and Their Degradation Characteristics

Complementing the keyword evolution analysis, microbial taxa were ranked by their occurrence frequency within the established local literature corpus (Table 1). The analysis identified *P. aeruginosa*, *B. subtilis*, and *R. erythropolis* as increasingly prominent research subjects [10,11,12], characterized by their capacity to produce biosurfactants and degrade complex petroleum hydrocarbons, including polycyclic aromatic compounds and diverse alkanes. Similarly, *C. daeguensis*, *A. baumannii*, and *D. lutea* have garnered growing scholarly attention owing to their exceptional environmental adaptability—specifically salinity tolerance and heavy metal resistance—coupled with robust survival capabilities [13,14,15].

#### 3.1.3. Inter-Genus Co-Occurrence Network Derived from Literature-Reported Degradation Consortia

**Figure 2:**
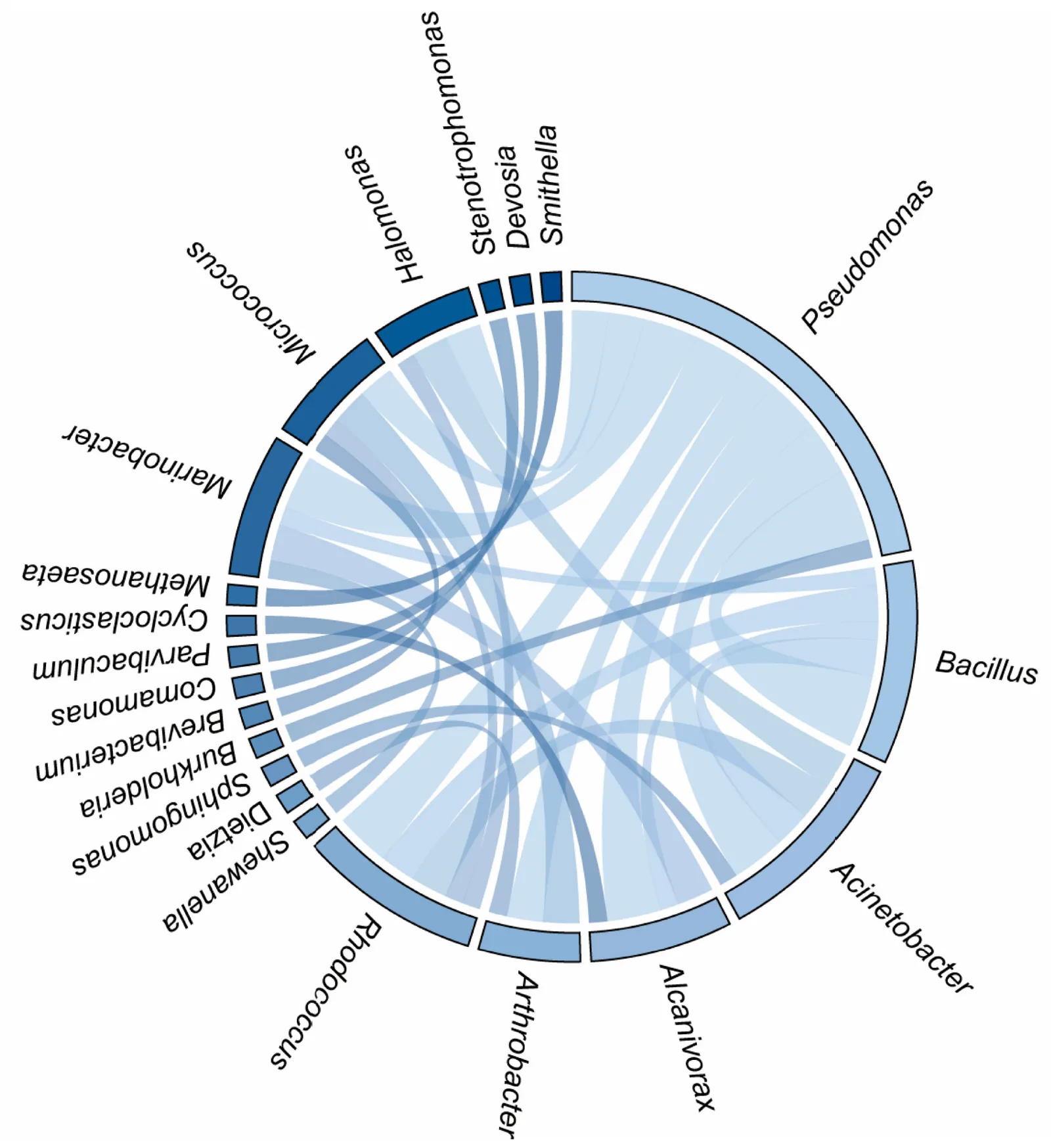
Chord diagram of microbial co-occurrence relationships among core petroleum-degrading taxa. The diagram visualizes the pairwise co-occurrence frequencies of the top 30 dominant species extracted from petroleum hydrocarbon-degrading consortia in 100 highly cited studies on petroleum pollution remediation. The width of the ribbons (chords) connecting the taxa is proportional to their co-occurrence frequency, reflecting the strength of their associative relationships.

A microbial co-occurrence network was constructed by systematically retrieving pairwise associations among bacterial taxa from published studies on petroleum-degrading microbial consortia. Specifically, we extracted taxa that were documented together in actual mixed cultures (or natural consortia) designed for hydrocarbon degradation. The resulting chord diagram (Figure 2) visualises the frequency and strength of literature-reported co-occurrence relationships among the genera represented by the 15 candidate strains. The network clearly reveals that *Pseudomonas*, *Bacillus*, and *Acinetobacter* constitute the core dominant genera, exhibiting extensive connectivity with a wide range of microbial taxa within reported degradation consortia.

Guided by these quantitative rankings and consortium-derived co-occurrence patterns, we prioritized the selection of ecologically representative type species, specifically *P. aeruginosa* and *B. subtilis* as well as others, and selected representative strains for comparative genomic analysis. In contrast, *Pseudomonas putida* and *Bacillus cereus* were excluded from the final list of strains subjected to further analysis.

### 3.2. Comparison of Distribution Patterns of Abundant Petroleum Hydrocarbon Degradation Genes in Different Genera

**Figure 3:**
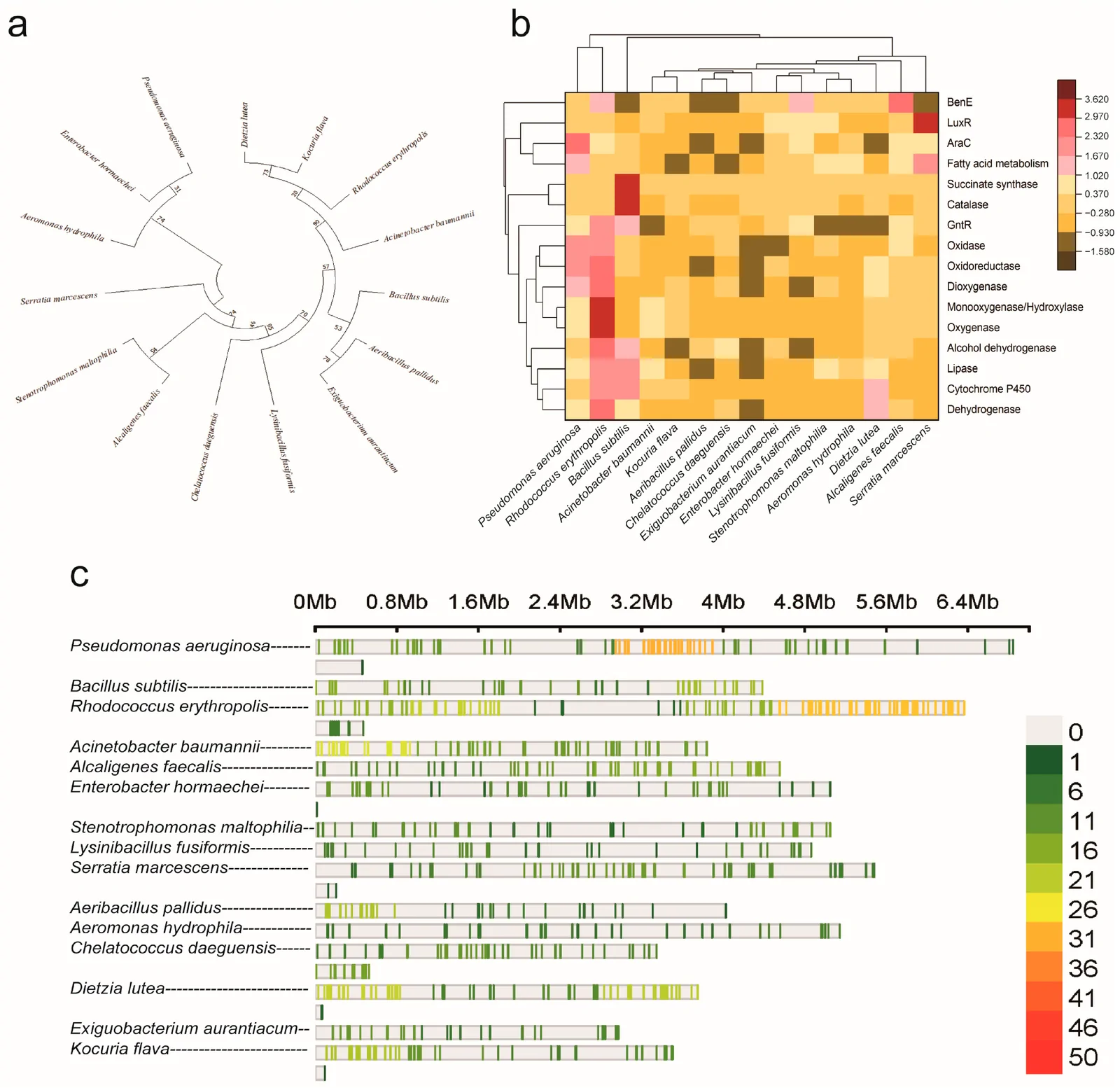
Comparative analysis of the genomic architecture of 15 petroleum hydrocarbondegrading bacteria. (**a**) Phylogenetic tree illustrating the evolutionary relationships among the 15 selected bacterial strains; (**b**) Heatmap showing the abundance of genes associated with direct degradation enzyme systems; (**c**) Chromosomal localization distribution of degradationrelated genes across the genomes of the 15 strains. Strain details are shown in Supplementary Table S1.

To systematically elucidate the genetic determinants underpinning the observed degradation phenotypes, this study conducted a comprehensive comparative genomic analysis based on the high-quality, chromosome-level assemblies of the selected fifteen strains, further integrated with functional annotations curated from multiple authoritative databases. Phylogenetic reconstruction based on core orthologous genes (Figure 3a) revealed clear evolutionary divergence among petroleum hydrocarbon degraders: genera such as *Pseudomonas* exhibited a relatively high degree of evolutionary conservation, whereas genera such as *Alcanivorax* displayed notably varied evolutionary trajectories. Subsequent heatmap profiling delineating the abundance of degradation enzyme coding genes (Figure 3b) showed that *P. aeruginosa* RW109 and *R. erythropolis* X5 harbored comparatively broader genomic repertoires for hydrocarbon catabolism, while *B. subtilis* PRO122 was distinctly enriched in catalase-coding gene families [19]. The genomic distribution mapping of degradation determinants (Figure 3c) demonstrated that these functionally relevant genes were preferentially localized within the genomic architectures of *P. aeruginosa* RW109 and *B. subtilis* PRO122, largely confined to discrete chromosomal loci or resident on conjugative plasmids. These spatially organized clusters encompassed genes encoding key biotransformation enzymes—including hydroxylases, epoxidases, and biosurfactant synthases—as well as associated regulatory elements.

### 3.3. The Potential Complementarity in Petroleum Hydrocarbondegradation Is Reflected by Accessory Genes of Distinct Strains Across Genera

**Figure 4:**
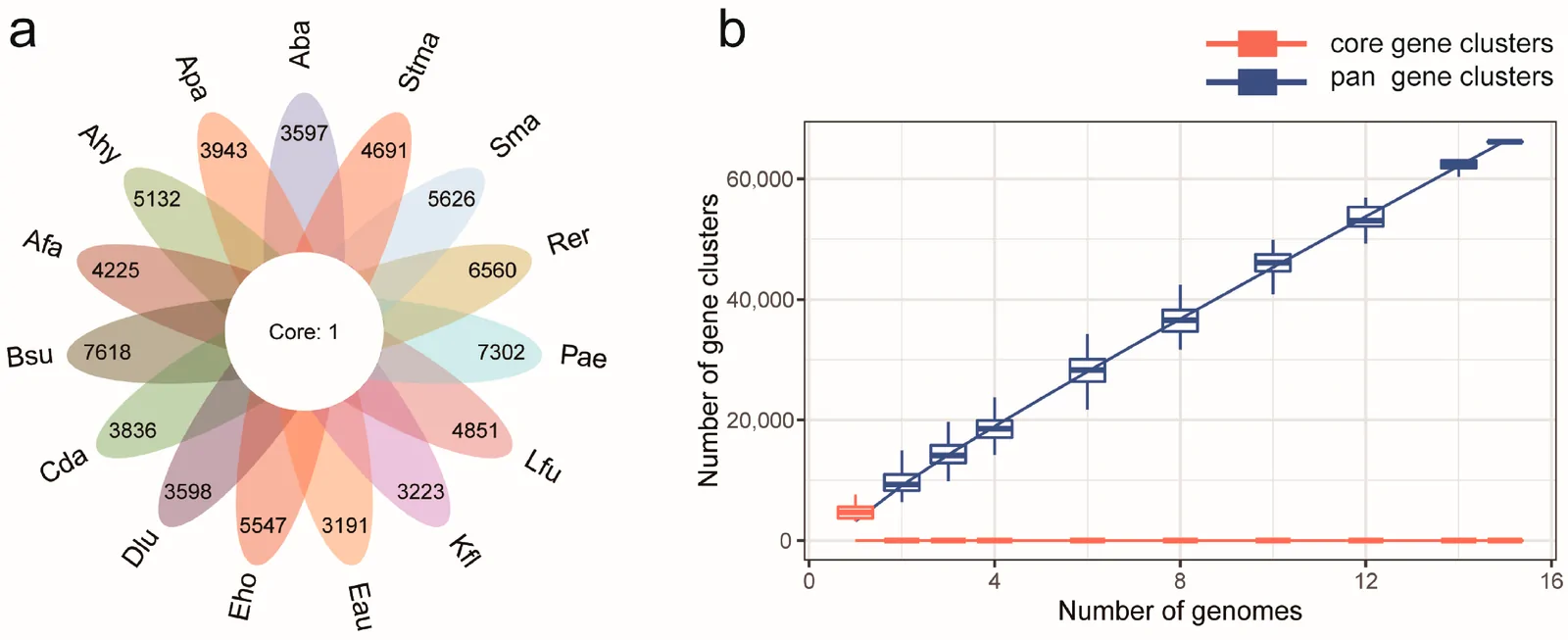
Comparative pan-genome analysis of 15 bacterial strains from different genera. (**a**) Gene flower plot (Venn diagram) illustrating the distribution of orthologous gene clusters. Each petal represents a single strain. The number of orthologous gene clusters is displayed in the center, while the number of strain-specific genes is indicated on the outer side of each petal. Strain abbreviations are derived from the first letter of the genus name and the first two letters of the species name. Abbreviations: *Pseudomonas aeruginosa* RW109 (Pae), *Bacillus subtilis* PRO122 (Bsu), *Rhodococcus erythropolis* X5 (Rer), *Acinetobacter baumannii* AC1633 (Aba), *Alcaligenes faecalis* NY11312 (Afa), *Enterobacter hormaechei* MRSN791417 (Eho), *Stenotrophomonas maltophilia* SM866 (Stma), *Lysinibacillus fusiformis* NEB1292 (Lfu), *Serratia marcescens* CM2015_854 (Sma), *Aeribacillus pallidus* KCTC3564 (Apa), *Aeromonas hydrophila* K533 (Ahy), *Chelatococcus daeguensis* TAD1 (Cda), *Dietzia lutea* YIM 80766 (Dlu), *Exiguobacterium aurantiacum* CX2 (Eau), *Kocuria flava* HO-9041 (Kfl). Core: 1” indicates that the number of genes in the core genome is 1. (**b**) Pan-genome and core genome curves for the 15 strains. The blue line represents the pan-genome size, and the orange-red line represents the core genome size.

To comprehensively evaluate the genomic diversity among the 15 selected strains, this study delineated their core and accessory gene sets based on orthologous clustering and visualized the overlap using a Venn diagram (Figure 4a). A rigorous comparative analysis revealed that only a single orthologous gene cluster was ubiquitously conserved across all fifteen microorganisms, accounting for a mere 0.13‰ of the total gene clusters identified. Quantitatively, the average gene complement per strain was 4864, exhibiting significant inter-strain variability; *E*. *aurantiacum* CX2 possessed the most streamlined genomic architecture with 3192 genes, whereas *B*. *subtilis* PRO122 harbored the most expansive repertoire with 7619 genes. Correspondingly, the number of accessory genes per strain ranged extensively from 3191 to 7618. To further illuminate the genomic fluidity across these phylogenetically diverse bacteria, the pan-genome accumulation curve was constructed (Figure 4b). The trajectory of the pan-genome exhibited a characteristic and sustained expansion as additional strains were incorporated. Specifically, the cumulative size of the pan-genome burgeoned at an average increment of 5709 novel genes per strain during the integration of the initial six genomes, subsequently decelerating to an average accretion of 3543 genes per strain as the remaining nine genomes were assimilated, ultimately culminating in a vast repository of 66,147 discrete gene clusters.

**Figure 5:**
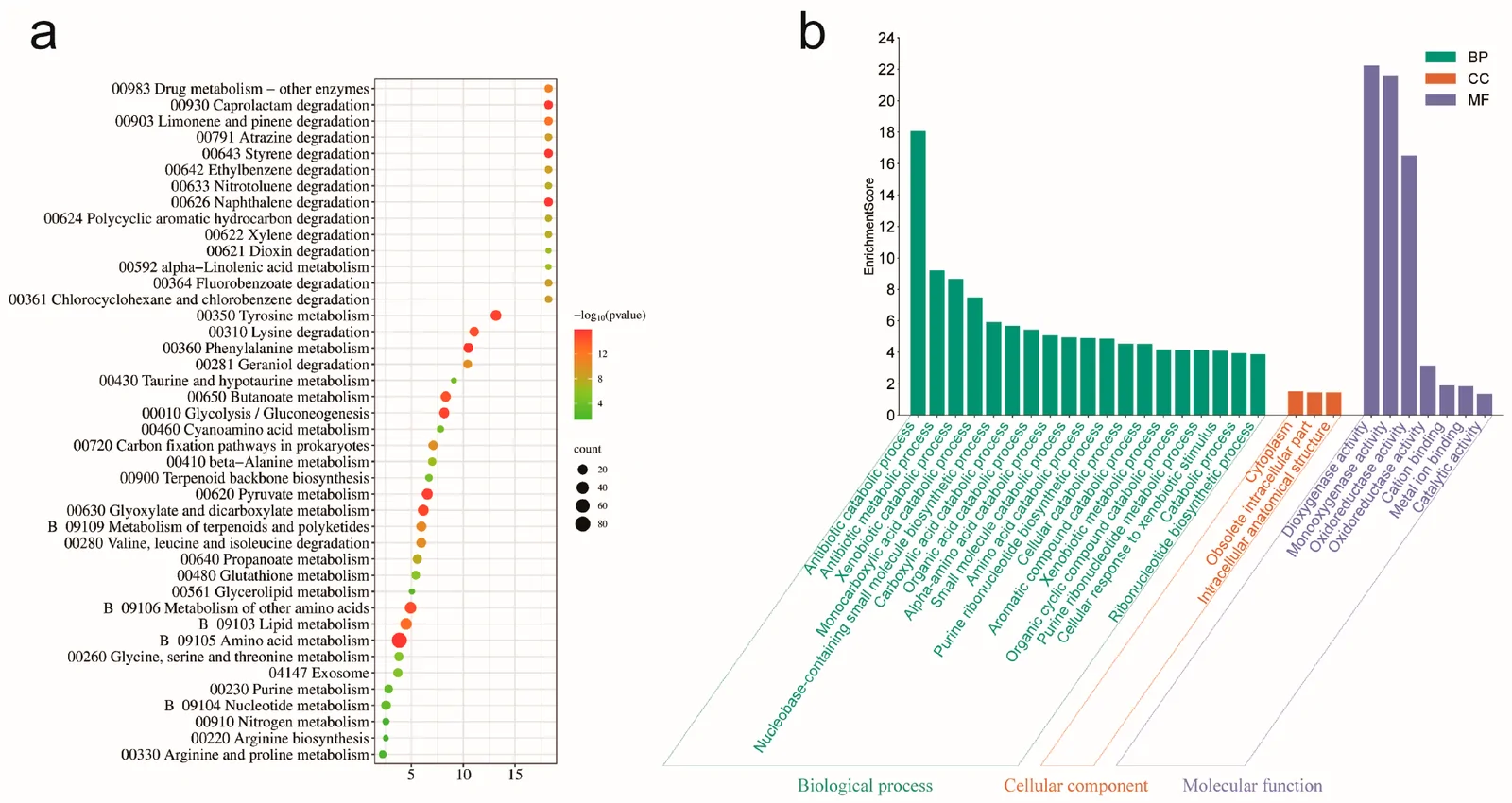
Enrichment analysis of variable genes across the 15 strains. (**a**) KEGG pathway enrichment analysis of the variable gene sets. (**b**) Gene Ontology (GO) enrichment analysis of the variable gene sets.

Enrichment analyses (Figure 5) revealed that genes critically involved in hydrocarbon catabolism—including those encoding dioxygenases, oxidoreductases, and monooxygenases—were predominantly located in the accessory genome rather than the core genome. In addition to differences in gene content, degradation gene clusters residing in the accessory genome were frequently accompanied by dedicated regulatory elements, such as two-component signaling systems. This genomic organization allows individual strains to dynamically adjust the transcriptional prioritization of specific degradation pathways in response to variations in the composition of environmental hydrocarbons, thereby contributing to coordinated metabolic regulation at the population level.

### 3.4. Synergistic Effects of Microbes from Different Genera in Addressing Recalcitrant Petroleum Hydrocarbon Components

**Figure 6:**
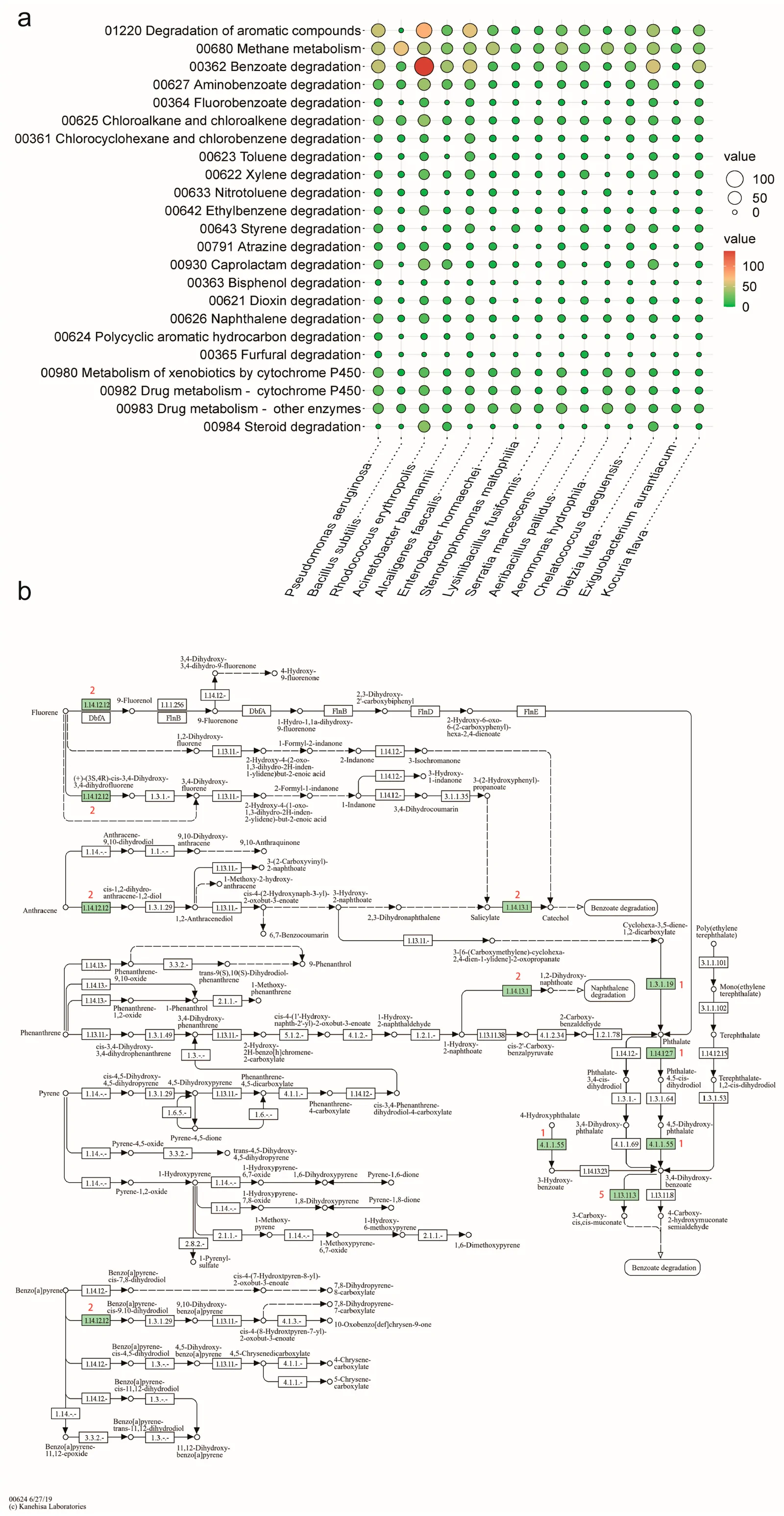
Complementarity analysis of degradation pathways among the 15 selected strains. (**a**) Stacked bubble chart illustrating the enrichment of degradation-related functional genes across the 15 strains. (**b**) Schematic diagram of the KEGG PAH degradation pathway (map00624). The red numbers indicate the count of microbial strains possessing genes annotated to that specific step in the pathway.

To comprehensively characterize the genomic diversity and functional complementarity among the 15 selected strains, this study examined the genetic potential and division of labor among different microorganisms across key nodes of the PAH degradation pathway. As shown in Figure 6a, the profile of significantly enriched functional genes within hydrocarbon degradation pathways revealed substantial inter-strain variability. Benzoate degradation, methane metabolism, and aromatic compound degradation represented the three most enriched pathways, with distinct strains exhibiting specific distribution patterns. *P. aeruginosa* harbored the highest number of enriched genes in PAH degradation (map00624), methane metabolism (map00680), and aromatic compound degradation (map01220). *R. erythropolis* X5 exhibited the highest gene counts in benzoate and general aromatic degradation, whereas *A. baumannii* AC1633 contained a comparatively lower number of annotated genes. *S. maltophilia* SM866, *L. fusiformis* NEB1292, and *E. aurantiacum* CX2 displayed relatively low gene counts across most metabolic pathways examined. *S. marcescens* CM2015_854 showed a notably high abundance of genes specifically within the benzoate degradation pathway (map00362). A targeted genomic analysis of the PAH degradation pathway (Figure 6b) revealed that only six strains—*P. aeruginosa*, *R. erythropolis*, *A. baumannii*, *A. faecalis*, *C. daeguensis*, and *K. flava*—carried the requisite catabolic genes. Genomic mapping further indicated that these strains collectively encoded a complete set of enzymatic cascades within the KEGG PAH degradation module (map00624). In the corresponding heatmap (Figure 6b), these six strains were characterized by dense distributions of green blocks and correspondingly low numerical indices.

## 4. Discussion

The diachronic evolution of keywords reveals profound paradigm shifts within the field. The persistent top ranking of “Community/Consortium” underscores the growing recognition of microbial synergy and diversity as critical determinants of bioremediation efficacy. The declining frequencies of “16S rRNA/16S rDNA” and “Pseudomonas” reflect a transition from mere taxonomic identification toward functional and mechanistic investigations, while the notably high frequency of “Pseudomonas aeruginosa” cements its status as a premier model organism in hydrocarbon remediation. The emergence of “Plant/Phytoremediation” and “Rhizosphere/Rhizobacteria” signals the increasing integration of plant growth-promoting rhizobacteria into phytoremediation strategies. Concurrently, the prominence of “PAH” and “Phenanthrene” indicates a research deepening into recalcitrant components, particularly polycyclic aromatic hydrocarbons. The high ranking of “Gene/Expression” alongside multi-omics methodological terms illustrates the pervasive integration of these advanced technologies. Such technological convergence provides a robust foundation for “bottom-up” consortium design: the acquisition of complete genome sequences, metagenomic maps, and gene expression profiles enables systematic dissection of degradation pathways, regulatory networks, and enzymatic mechanisms; these panoramic genomic insights are crucial for deciphering the metabolic traits and interspecies complementarity of candidate strains, thereby facilitating the rational assembly of synthetic consortia.

The ranking of microbial taxa according to mention frequency reveals that biosurfactant-producing species (*P. aeruginosa*, *B*. *subtilis*, *R*. *erythropolis*) and environmentally robust species (*C*. *daeguensis*, *A*. *baumannii*, *D*. *lutea*) have become focal research subjects, reflecting the field’s dual emphasis on degradation efficiency and environmental adaptability. When constructing synthetic microbial communities, targeted combinations of different strains can expand or precisely tailor the degradation spectrum of dominant petroleum hydrocarbon-degrading bacteria, thereby maximizing respective advantages while compensating for inherent limitations. Although *P. putida* and *B. cereus* were not included in the priority panel for comparative genomic analysis in this study, they hold considerable theoretical importance in hydrocarbon bioremediation: *P. putida* harbors a highly conserved and efficient *alk* gene cluster encoding an alkane hydroxylase system capable of specifically oxidizing medium- to long-chain *n*-alkanes [25], while the abundance of *xyl* and *tod* operons within its genome confers robust meta- and ortho-cleavage metabolic capabilities against monocyclic aromatic hydrocarbons such as toluene and xylene, channeling aromatic intermediates into the TCA cycle [26]; concerning PAHs, its cytochrome P450 monooxygenase system plays a pivotal role in the initial hydroxylation of low-concentration PAHs [27]. On the other hand, *B. cereus*, representing Gram-positive bacteria, exhibits distinct environmental adaptability and catabolic versatility: studies indicate that it significantly enhances the bioavailability of hydrophobic hydrocarbons by secreting biosurfactants that reduce interfacial tension at the oil–water interface [28], carries diverse dehydrogenase and oxygenase genes enabling effective mineralization of aliphatic hydrocarbons and certain low-molecular-weight PAHs [29], and possesses physiological resilience to saline and pH-fluctuating conditions that underscores its potential for bioremediation in extreme environments [29]. Nevertheless, the metabolic profiles of *P. putida* and *B. cereus* provide crucial supplementary perspectives for deciphering the complexity of hydrocarbon degradation networks, and their potential for synergistic degradation warrants further exploration in the future design of broad-spectrum remediation consortia. The co-occurrence chord diagram identifying *Pseudomonas*, *Bacillus*, and *Acinetobacter* as core dominant genera suggests that these groups serve as pivotal players in sustaining high-efficiency degradation within the consortium, likely achieving spatiotemporal complementarity through mechanisms such as interspecies electron transfer and functional redundancy, thereby ensuring the stability of remediation functions under complex contamination scenarios [30]. However, it must be acknowledged that these co-occurrence patterns arise from bibliometric analyses and are thus inevitably influenced by biases stemming from different experimental designs and cannot be taken as direct evidence of in situ biological interactions. Consequently, the network presented herein serves primarily as a hypothesis-generating tool to guide the rational assembly of synthetic consortia, necessitating empirical validation through subsequent co-culture experiments. The significant divergence in degradation capabilities and stress tolerance traits across different strains enables the targeted assembly of high-performance degrader consortia, as exemplified by the tailored assemblages identified in existing research (e.g., *Alcanivorax*–*Pseudomonas* for aliphatics and PAHs [31]; *Methyloversatilis* and *Thauera* for monoaromatics [32,33]; *A*. *baumannii* and *A*. *faecalis* for stress resistance and structural stability [34]), with algorithm-driven optimization of inoculation ratios further facilitating the construction of high-efficacy synthetic degrader communities [35].

The biogeochemical process of petroleum hydrocarbon biodegradation constitutes a sophisticated and hierarchically organized cascade, governed by the synergistic orchestration of multiple enzymatic complexes and the stringent spatiotemporal regulation of metabolic networks, with its biochemical essence residing in the stepwise mineralization of recalcitrant hydrocarbons via phylogenetically distinct microbial pathways [1]. Aerobic hydrocarbonoclastic bacteria predominantly rely on sophisticated oxygenase systems to initiate catabolic reactions: flavin-dependent monooxygenases (exemplified by certain isoforms in *Streptomyces*) or evolutionarily refined dioxygenases (such as those characterizing *P. aeruginosa*) facilitate the incorporation of molecular oxygen atoms into inert hydrocarbon substrates, thereby generating reactive intermediates including aliphatic alcohols and aldehydes, which are subsequently channeled into the central tricarboxylic acid (TCA) cycle to achieve complete oxidative decomposition into CO_2_ and H_2_O [36,37]. In stark contrast, obligate anaerobic bacteria (typified by *Geobacter* spp.) employ adenosine triphosphate (ATP)-dependent CoA ligases to activate hydrocarbon moieties under anoxic conditions; these activated thioester intermediates subsequently undergo iterative cycles of β-oxidation to yield acetyl-CoA, a process intricately coupled with the reduction of exogenous electron acceptors such as sulfate or ferric iron to harvest bioenergetic currency. Although this anaerobic catabolic route is inherently constrained by slower kinetic rates, it remains physiologically indispensable for driving bioremediation processes within oligotrophic and anoxic deep-subsurface environments [38,39]. Concomitantly, anoxygenic phototrophic microorganisms harness exogenous light energy to fuel specialized electron transport chains, thereby powering the thermodynamically unfavorable steps required for hydrocarbon bond cleavage. The efficacy and scope of the degradation process are fundamentally dictated by the substrate specificity and catalytic efficiency of the enzymatic systems encoded by functional gene cassettes. Naphthalene dioxygenase, operating via its dedicated ferredoxin–NADP^+^ reductase complex, not only catalyzes the stereospecific hydroxylation and subsequent ring cleavage of naphthalene but also exhibits remarkable promiscuity towards structurally complex polycyclic aromatic hydrocarbons, including high-molecular-weight substrates such as benzo[*a*]anthracene [37]. With respect to the catabolism of long-chain alkanes, alkane hydroxylases—including the membrane-bound AlkB and the soluble cytochrome P450 CYP153—collaborate intimately with redox cofactors (NADH/FMN) to facilitate terminal oxidation. For instance, the isoform AbAlkB in *Alcanivorax* exhibits exquisite substrate selectivity, preferentially targeting C_5_–C_15_ straight-chain alkanes [40], whereas representatives of the genus *Dietzia* achieve progressive carbon-chain shortening through a sophisticated division of labor between AlkB and CYP153 isoforms [41]. Furthermore, NAD(P)^+^-dependent alcohol dehydrogenases catalyze the critical oxidation step converting primary alcohols to ketones, thereby advancing the oxidative dismantling of alicyclic hydrocarbons such as cyclohexane [42]. Collectively, these intricate enzymatic systems—defined by precise substrate recognition pockets, regioselective oxidation mechanisms, and tightly coordinated coenzyme shuttles—construct a multi-tiered degradation architecture encompassing primary terminal oxidation, secondary ring decomposition, and ultimate complete mineralization. The observed nuanced functional differentiation among strains, as revealed by the phylogenetic and genomic analyses (Figure 3, Figure 4 and Figure 6), delineates a putative genomic blueprint for the rational bottom-up design of synthetic microbial consortia, as empirically illustrated by the comprehensive gene–enzyme mapping presented in Table 2. The extensive genomic repertoires of *P. aeruginosa* RW109 and *R. erythropolis* X5 align with prior empirical investigations demonstrating that synergistic consortia formulated from these two genera can achieve crude oil and isoprenoid alkane degradation efficiencies as high as 92% [43], substantiating the predictive value of genomic potential analysis. Conversely, the distinct enrichment of catalase-coding gene families in *B. subtilis* PRO122 underscores its formidable physiological adaptability and resilience against oxidative environmental stressors frequently encountered in contaminated habitats [19]. The dense clustering of degradation determinants within discrete chromosomal loci or conjugative plasmids in *P. aeruginosa* RW109 and *B. subtilis* PRO122 suggests that such spatial organization likely facilitates coordinated transcriptional activation and translational regulation of these pivotal gene cassettes, thereby optimizing metabolic flux. For instance, engineered synthetic consortia constructed based on the strategic complementarity of these pivotal genetic modules have been empirically demonstrated to successfully activate core catabolic operons, notably *alkB* and *pcaH*, resulting in a significantly broadened substrate spectrum for the assimilation of recalcitrant long-chain alkanes. Nevertheless, rigorous empirical validation through meticulously controlled co-culture experiments remains an imperative prerequisite to confirm and quantify the predicted consortium-level catabolic performance. Collectively, these findings not only illuminate the spatial organization of catabolic potential but also highlight specific target gene clusters for future genetic engineering interventions aimed at augmenting the efficacy of environmental bioremediation strategies.

The extreme paucity of shared genetic material—with only a single orthologous gene cluster conserved across all fifteen strains, accounting for a mere 0.13‰ of the total gene clusters identified—underscores the vast phylogenetic distances separating these taxa. The substantial inter-strain variability in gene complements (ranging from 3192 in *E. aurantiacum* CX2 to 7619 in *B. subtilis* PRO122) and the extensive range of accessory genes (3191 to 7618) further highlight the individualized genomic signatures within the assemblage. The sustained upward trajectory of the pan-genome accumulation curve, devoid of any discernible plateau, provides unequivocal evidence that the pan-genome remains definitively “open.” This expansive genomic architecture, coupled with the exceptionally low proportion of core genes, signifies an extraordinary degree of genetic heterogeneity. Such profound genomic diversity is intrinsically linked to the deliberate selection strategy employed, which prioritized a rich phylogenetic tapestry of genera characterized by inherently poor sequence homology, thereby capturing the full spectrum of adaptive solutions to hydrocarbon catabolism. The predominant localization of degradation-related genes—including dioxygenases, oxidoreductases, and monooxygenases—within the accessory rather than the core genome (Figure 5) aligns closely with the widely substantiated role of oxygenases and oxidoreductases as central catalytic drivers in petroleum hydrocarbon biodegradation, as independently corroborated by both controlled experimental studies and large-scale meta-analyses [36,37,42]. From the perspective of genome plasticity, the conspicuous prevalence of degradation determinants within the accessory genome strongly implies a heightened propensity for horizontal gene transfer (HGT). In principle, such genetic mobility could facilitate the inter-taxonomic dissemination of catabolic functions, thereby contributing to microbial adaptation under hydrocarbon-impacted or otherwise environmentally stressful conditions [44,45]. Prior studies have demonstrated that microbial strains can readily acquire supplementary degradation determinants via plasmid-mediated conjugation or natural transformation, lending plausibility to a putative “relay” model wherein stepwise hydrocarbon catabolism is achieved through coordinated gene exchange within microbial consortia. Illustrative examples include the plasmid-mediated 2,4-dinitrotoluene degradation capability of *Arthrobacter* sp. K1 [46], and the acquisition of diethyl phthalate catabolism by *P. putida* through plasmid-mediated gene exchange [47]. Whether such plasmid-borne functional complementarity operates among the fifteen strains examined herein warrants rigorous experimental scrutiny in future work. Beyond mere gene content, degradation gene clusters nested within the accessory genome frequently co-evolve with sophisticated regulatory elements, such as two-component signaling systems. This genetic architecture enables different strains to dynamically modulate the transcriptional priority of specific degradation pathways in response to fluctuations in the compositional profile of environmental hydrocarbons, thereby fostering metabolic regulatory synergy at the population level. For instance, synergistic interactions within the defined bacterial consortium OPK—comprising *Mycolicibacterium* strains PO1 and PO2, *Novosphingobium pentaromativorans* PY1, and *B. subtilis* FW1—facilitate the efficient degradation of pyrene across a wide spectrum of pH, temperature, and salinity regimes, and even in the presence of competing polycyclic aromatic hydrocarbons [48]. Given that the accessory genome harbors highly differentiated degradation modules, microbial communities are theoretically well-positioned to achieve comprehensive coverage of complex metabolic networks through an intricate “division of labor and cooperation.” This functional stratification significantly enhances the collective adaptability of the consortium when confronting the extreme physicochemical heterogeneity characteristic of petroleum-contaminated environments, as further contextualized by the enrichment profiles depicted in Figure 5. Nevertheless, the actual frequencies and net ecological consequences of HGT events remain to be quantitatively validated through in situ measurements.

The intersectionality and inherent plasticity of microbial metabolic pathways constitute the molecular bedrock for deciphering the mechanistic intricacies of petroleum hydrocarbon biodegradation. During in situ bioremediation, the catabolic limitations of a single isolate are frequently circumvented by the intersecting cyclic metabolic fluxes established among syntrophic microbial species. A pertinent example is the obligate syntrophy observed between *P. stutzeri* SLG510A3-8 and *Dietzia* sp. DQ12-45-1b; despite the former’s inability to utilize hexadecane (C16) as a sole carbon source, it effectively exploits the intermediate metabolites excreted by the latter to achieve synergistic degradation [49]. Such spatiotemporal complementarity in metabolic circuitry effectively mitigates the cytotoxic accumulation of intermediary compounds. Furthermore, the existence of synergistic interactions is often corroborated by the identification of complementary gene expression patterns across co-cultured taxa [50]. The heterogeneous distribution of catabolic genes across the 15 strains, as revealed in Figure 6a, underscores the vast diversity in environmental adaptation and pollutant degradation strategies. The identification of benzoate degradation, methane metabolism, and aromatic compound degradation as the three most significantly enriched pathways is consistent with both the bulk composition of crude oil and the established catabolic repertoires of environmental microbes. The distinct pathway distributions observed among strains suggest that *P. aeruginosa* RW109 possesses preeminent metabolic capacity across multiple degradation domains, while *R. erythropolis* X5 shows specialization in benzoate and general aromatic degradation. Although *A. baumannii* AC1633 possesses a comparatively lower gene count, it remains a critical player in the catabolism of specific pollutants such as xylene [51]. The disproportionately high abundance of benzoate degradation genes in *S. marcescens* CM2015_854 aligns with genomic evidence of a protocatechuate *para*-degradation pathway [52]. The focused genomic interrogation revealing that only six strains possess the requisite genetic determinants for the PAH degradation pathway, and these strains collectively encoded a complete set of enzymatic cascades within the KEGG PAH degradation module, implies a substantial potential for metabolic complementarity among these taxa. The dense distribution of green blocks and small numerical indices within the heatmap for these six strains (Figure 6b) further supports this interpretation. Empirical studies lend credence to this genomic inference; for instance, the P&A bacterial consortium has been documented to achieve average degradation rates exceeding 80% for naphthalene, phenanthren, and pyrene in oil-contaminated sludge [53]. Similarly, a solid composite bacterial agent engineered using *Luteimonas huabeiensis*, *C. daeguensis*, *P. aeruginosa*, and *B. subtilis* successfully degraded 96.6% of total petroleum hydrocarbons [54].

Beyond genomic complementarity, a rigorous evaluation of interspecific compatibility is a prerequisite for the rational construction of high-efficiency synthetic microbial consortia, specifically addressing potential antagonistic interactions and physiological mismatches among constituent strains. A primary challenge in consortium design is the suppression of specific strains by others through the production of antimicrobial compounds or intense resource competition. Although *P. aeruginosa* has been demonstrated to harbor a substantial repertoire of non-ribosomal peptide synthetase and polyketide synthase gene clusters, these are primarily associated with siderophore (pyoverdine) biosynthesis and biofilm formation, rather than encoding broad-spectrum bacteriocins targeting other degraders within the microbial community. *B. subtilis*, known for producing lipopeptides such as surfactin, exhibits antimicrobial activity primarily against phytopathogens and Gram-positive competitors; however, its interaction with Gram-negative genera like *Pseudomonas* and *Acinetobacter* is often characterized by synergistic biofilm enhancement rather than inhibition [25]. Furthermore, *R. erythropolis* lacks significant biosynthetic gene clusters for narrow-spectrum antibiotics targeting these specific co-members, thereby reducing the risk of intra-consortium toxicity [27]. Co-occurrence network analysis further supports this compatibility, demonstrating that *Pseudomonas*, *Rhodococcus*, and *Acinetobacter* frequently co-exist in natural hydrocarbon-contaminated environments, suggesting evolved mechanisms for niche partitioning and metabolic cross-feeding that mitigate direct antagonism [26]. Regarding physiological compatibility, the successful co-cultivation of these strains relies on overlapping tolerance ranges for temperature, salinity, and pH. The selected strains exhibit remarkable adaptability to mesophilic conditions (20–37 °C), which aligns with typical temperate soil and marine environments [29]. *P. aeruginosa* and *R. erythropolis* demonstrate broad pH tolerance (pH 5–9) and moderate halotolerance (up to 3–5% NaCl), ensuring stability under fluctuating environmental stresses [27]. *B. subtilis* contributes resilience through spore formation, allowing survival during transient adverse conditions, while maintaining active degradation at neutral pH and moderate salinity. The convergence of these physiological traits ensures that no single strain is excluded due to environmental mismatch, thereby facilitating sustained synergistic degradation of PAHs and aliphatic hydrocarbons. This multi-layered compatibility—genomic, ecological, and physiological—validates the rational selection of these fifteen strains for high-efficiency bioremediation applications, moving beyond empirical mixing to a knowledge-driven design strategy.

## 5. Conclusions

Constructing synthetic consortia represents a current frontier in petroleum hydrocarbon bioremediation research. With the rapid advancement of genomics and metagenomics technologies, investigations into hydrocarbon-degrading strains have progressively shifted toward elucidating gene-level regulatory mechanisms and mining novel functional determinants. A central scientific challenge lies in how to holistically integrate biodegradation phenotypes and cooperative interactions among disparate microorganisms to optimise strain selection and consortium architecture. To address this, we innovatively extrapolated the pan-genome concept from single-species analyses to encompass multiple genera, integrating bibliometric screening with comparative genomics to delineate the degradation characteristics of 15 representative strains at the molecular level.

Our multi-perspective analysis revealed that degradation traits exhibit both conservation and divergence across distinct patterns. Variability in degradation-associated gene quantity reflects a potential for metabolic division of labour, while dense clustering of these genes within chromosomes or plasmids facilitates coordinated expression. Their predominance within the accessory genome highlights evolutionary pressures shaping niche-specific catabolic functions, and enzymatic complements from different strains collectively saturate the nodes of PAH degradation pathways. Collectively, the genomic evidence supports a model wherein intergeneric cooperation alleviates the constraints of narrow substrate range and cytotoxic intermediate accumulation typically encountered in monocultures, while the inherent mobility of the accessory genome implies a capacity for dynamic network remodelling via horizontal gene transfer.

Nevertheless, several limitations warrant careful consideration. Our comparative genomics approach is inherently correlative; actual expression levels, enzyme activities, and metabolic stoichiometry require empirical validation through transcriptomic, proteomic, and metabolomic analyses. The predictive power of our model also depends on the completeness of current databases, and the influence of mobile genetic elements, epigenetic regulation, and complex abiotic factors on consortium efficacy in field settings remains insufficiently addressed. Despite these constraints, this study provides a genomic-level framework for the rational design of synthetic hydrocarbon-degrading consortia, demonstrating that systematic cross-generic comparative genomics combined with literature-derived co-occurrence analysis can effectively guide strain prioritisation and the prediction of metabolic complementarity, thereby establishing a theoretical foundation for knowledge-driven petroleum bioremediation strategies.:

## Figures and Tables

**Table 1:** Occurrence frequency and degradation characteristics of petroleum hydrocarbon degrading bacteria in the literature.

Species	Frequency Ranking	Score	Representative Literature with High Cited Scores	Degradation Capacity Description
*Pseudomonas aeruginosa*	1	0.071	Petroleum hydrocarbon degrading bacteria remediate oil pollution under aerobic conditions [10].	Efficiently degrade alkanes and produce surfactants.
*Bacillus subtilis*	2	0.052	A critical review of the analysis, purification and characterization of biosurfactants using rhamnolipids as a model [11].	Degrades a wide variety of petroleum hydrocarbons, including alkanes, aromatic hydrocarbons, and polycyclic aromatic hydrocarbons.
*Rhodococcus erythropolis*	3	0.100	Effectiveness and limitations of bioaugmentation and biostimulation processes, and applications to in situ remediation [12].	Good at degrading various alkanes, aromatic hydrocarbons and their derivatives, yields highly effective biosurfactants.
*Serratia marcescens*	4	0.014	Enhanced bioavailability and microbial biodegradation of polystyrene from the gut microbiome enriched from *Tenebrio molitor* [16].	Efficient degradation of aromatic hydrocarbons, especially polycyclic aromatic hydrocarbons.
*Acinetobacter baumannii*	5	0.013	Biodegradation studies were carried out by using a bacterial community consisting of *Bacillus subtilis*, *Pseudomonas stutzeri* and *Acinetobacter baumannii* [15].	Mainly degrades aromatic hydrocarbons; shows strong survival and degradation ability in complex environments.
*Stenotrophomonas maltophilia*	6	0.035	Synergistic ex situ biodegradation of crude oil by indigenous community of salt-tolerant bacteria [17].	Degrades a wide range of complex organic compounds, including alkanes and aromatic hydrocarbons.
*Dietzia lutea*	7	0.013	Efficient degradation of alkanes and crude oil by the salt-tolerant bacterium *Dietzia* strain CN-3 [13].	Degradation of various aromatic hydrocarbons and organic solvents.
*Alcaligenes faecalis*	8	0.005	Genomic and physiological signatures of *Alcaligenes aquatilis* QD168 reveal robust environmental adaptability [18].	Degrades a variety of aromatic hydrocarbons, alkanes and polycyclic aromatic hydrocarbons, and can carry out complex biotransformation.
*Lysinibacillus fusiformis*	9	0.008	Enhanced biodegradation of styrene vapor in a bio-trickling filter inoculated with biosurfactant-producing bacteria under hydrogen peroxide stimulation [19].	Producing a variety of enzymes and biosurfactants; degrades a wide variety of alkanes and aliphatic compounds.
*Enterobacter hormaechei*	10	0.009	Effects of petroleum-degrading bacteria competing with indigenous composting microorganisms on the bioremediation efficiency of petroleum sludge [20].	Degradation of polycyclic aromatic hydrocarbons and reduction of metal ions
*Aeromonas hydrophila*	11	0.003	Novel kinetic model development based on microbiome and biochar for in situ remediation of total petroleum hydrocarbons (TPHs) contaminated soils [21].	Degrading alkanes; Salt tolerance
*Exiguobacterium aurantiacum*	12	0.002	Potential of indole-3-acetic acid-producing rhizosphere bacteria to resist lead toxicity in oil-contaminated soils [22].	Degradation of multiple organic compounds in soil and water.
*Aeribacillus pallidus*	13	0.014	Degradation of mixed PAHs by pure and mixed cultures of biosurfactants produced by thermophilic and thermotolerant bacteria [23].	Degrades a variety of organic pollutants, including polycyclic aromatic hydrocarbons.
*Kocuria flava*	14	0.005	Catabolic enzyme activity during the biodegradation of tricyclic PAH by novel isolates [24].	Degradation of various alkanes and aromatic hydrocarbons in water bodies.
*Chelatococcus daeguensis*	15	0.007	Biodegradation of crude oil by *Chelatococcus daeguensis* HB-4 and its potential for microbial enhanced oil recovery (MEOR) in heavy oil reservoirs [14].	Degrade alkanes and aromatic hydrocarbons in high salinity environment.

Table Note: The highest citation score is defined as the maximum value of the ratio of the citation frequency of the literature associated with a specific strain to the number of articles published after its release.

**Table 2:** Encoding genes of microbial degradation enzyme systems and their metabolic functions.

Gene	Encoded Proteins	Functions
alkB	Alkane 1-monooxygenase	Oxidation of alkanes to corresponding alcohols
alkG	Rubredoxin-2	Further oxidation of an alcohol to an aldehyde or acid
alkT	Rubredoxin-NAD(+) reductase	Oxidation of aldehydes to acids
phnA	Phosphonoacetate hydrolase	Oxidation of phenol to catechol
phnB	Catechol dioxygenase	Further oxidation of catechol to cis-cis-dihydroxycyclohexenone
pahA	Naphthalene dioxygenase	Oxidation of naphthalene to 1, 2-dihydronaphthalene-1, 2-diol
pahB	1, 2-dihydronaphthalene-1, 2-diol dehydrogenase	Oxidation of 1, 2-dihydronaphthalene-1, 2-diol to catechol
katE	Catalase	Degrades hydrogen peroxide and alleviates oxidative stress
sodA	Superoxide dismutase	Scavenging superoxide radicals
hsp60	Heat shock protein	Helps proteins fold correctly and function restore
glnA	Glutamine synthetase	Involved in the assimilation metabolism of nitrogen
pstS	Phosphate transporter	Promote phosphorus uptake
xylE	Catechol dioxygenase	Conversion of catechol to 2-hydroxycis-cis-2, 4-pentadienoic acid
xylA	Benzoate dioxide dioxygenase	Conversion of benzoic acid to cis-cis-dihydroxy-1, 2-diphenylacetic acid
catA	Catechol 1, 2-dioxygenase	Cleavage of catechol to cis-cis-2-hydroxycis-cis-2, 4-pentadienoic acid
todC1C2BA	Toluene dioxygenase complex	Oxidation of toluene to catechol
nahA	Naphthalene dioxygenase	Oxidation of naphthalene to 1, 2-dihydronaphthalene-1, 2-diol
nahB	1, 2-dihydronaphthalene-1, 2-diol dehydrogenase	Oxidation of 1, 2-dihydronaphthalene-1, 2-diol to catechol
nahC	Catechol 1, 2-dioxygenase	Cleavage of catechol to cis-cis-2-hydroxycis-cis-2, 4-pentadienoic acid
xylM	Xylene monooxygenase	Oxidation of xylene to methylbenzyl alcohol
xylB	Methylbenzyl alcohol dehydrogenase	Oxidation of methyl benzyl alcohol to benzaldehyde
xylC	Benzaldehyde dehydrogenase	Oxidation of benzaldehyde to benzoic acid
benA	Benzoate 1,2-dioxygenase subunit alpha	Oxidation of benzene to phenol
benB	Benzoate 1,2-dioxygenase subunit beta	Oxidation of phenol to catechol
dmpN	Phenol 2-monooxygenase, oxygenase component DmpN	Oxidation of p-cresol to p-cresoldiphenol
dmpL	Phenol 2-monooxygenase, oxygenase component DmpL	Further oxidation of p-cresoldiol to cis-cis-2-hydroxycis-cis-2, 4-pentadienoic acid
rubB	Cytochrome P450 reductase	Provide reducing power for P450 enzyme system
rubA	Rubredoxin	Participates in oxidation reactions as part of the electron transport chain
cisD	Cis-cis-cyclohexene dioxygenase	Oxidation of cis-cis-cyclohexene to cis-cis-cyclohexenecyclohexanediol
cisC	Cis-cis-cyclohexenecyclohexanediol dehydrogenase	Oxidation of cis-cis-cyclohexenecyclohexanediol to cis-cis-cyclohexenecyclohexanol
todA	Toluene dioxidase	Oxidation of toluene to catechol
todB	Catechol dioxygenase	Oxidation of catechol to 1, 2-benzenediol
todD	Toluene dioxygenase	Oxidation of toluene to catechol
xylXY	Xylene dioxygenase	Oxidation of xylene to methylcatechol
xylDE	Catechol dioxygenase	Oxidation of catechol to 2-hydroxycis-cis-2, 4-pentadienoic acid
xylG	Methylcatechol dehydrogenase	Oxidation of methylcatechol to methylphenaldehyde
xylH	Methyl o-phenaldehyde dehydrogenase	Oxidation of methylphenaldehyde to methylphenolic acid
xylI	Phthalate dehydrogenase	Dehydrogenation of phthalic acid to phthalic acid
xylJKL	Phthalate dioxygenase	Oxidation of phthalic acid to phthalic diol
xylMNO	Phthalate diol dehydrogenase	Dehydrogenation of phthalic diol to phthalic acid

## Data Availability

The data supporting the findings of this study can be found in the relevant databases as specified in the Methods section.

## Supplementary Materials

The following supporting information can be downloaded at https://www.mdpi.com/article/10.3390/biology15161401/s1. Table S1: Genbank accession number and genome data acquisition address of petroleum hydrocarbon degrading bacteria strains.
