# Supplementary material for: Genomic Features, Functional Complementarity, and the Potential for Constructing Synthetic Consortia from Dominant Petroleum-Degrading Bacteria
Source: Biology (Basel). 2026 Aug 15;15(16):1401. doi: 10.3390/biology15161401 (PMC13510268; doi:10.3390/biology15161401)
Supplement: Supplementary file 1 [file biology-15-01401-s001.zip › biology-4454007-supplementary.pdf]

Table S1. Genbank accession number and genome data acquisition address of petroleum hydrocarbon degrading bacteria strains

Strains	GenBank	NCBI Link
Pseudomonas aeruginosa Isolate RW109	GCA_900243355.1	*Pseudomonas aeruginosa genome assembly RW109 - NCBI - NLM (nih.gov)*
Bacillus subtilis Strain PRO122	GCA_029762375.1	*Bacillus subtilis genome assembly ASM2976237v1 - NCBI - NLM (nih.gov)*
Rhodococcus erythropolis Strain X5	GCA_008728655.1	*Rhodococcus erythropolis genome assembly ASM872865v1 - NCBI - NLM (nih.gov)*
Acinetobacter baumannii Strain AC1633	GCA_014874575.1	*Acinetobacter baumannii genome assembly ASM1487457v1 - NCBI - NLM (nih.gov)*
Alcaligenes faecalis Strain NY11312	GCA_027595045.1	*Alcaligenes faecalis genome assembly ASM2759504v1 - NCBI - NLM (nih.gov)*
Enterobacter hormaechei (CDC Enteric Group 75) Strain MRSN791417	GCA_030717405.1	*Enterobacter hormaechei genome assembly ASM3071740v1 - NCBI - NLM (nih.gov)*
Stenotrophomonas maltophilia Strain SM 866	GCA_007833655.1	*Stenotrophomonas maltophilia genome assembly ASM783365v1 - NCBI - NLM (nih.gov)*
Lysinibacillus fusiformis Strain NEB1292	GCA_016925635.1	*Lysinibacillus fusiformis genome assembly ASM1692563v1 - NCBI - NLM (nih.gov)*
Serratia marcescens Strain CM2015_854	GCA_022439585.1	*Serratia marcescens genome assembly ASM2243958v1 - NCBI - NLM (nih.gov)*
Aeribacillus pallidus Strain KCTC3564	GCA_002243625.1	*Aeribacillus pallidus genome assembly ASM224362v1 - NCBI - NLM (nih.gov)*
Aeromonas hydrophila Strain K533	GCA_029025785.1	*Aeromonas hydrophila genome assembly ASM2902578v1 - NCBI - NLM (nih.gov)*

Chelatococcus daeguensis Strain TAD1	GCA_001887265.1	*Chelatococcus daeguensis genome assembly ASM188726v1 - NCBI - NLM (nih.gov)*
Dietzia lutea Strain YIM 80766	GCA_003096075.1	*Dietzia lutea genome assembly ASM309607v1 - NCBI - NLM (nih.gov)*
Exiguobacterium aurantiacum Strain CX2	GCA_024362205.1	*Exiguobacterium aurantiacum genome assembly ASM2436220v1 - NCBI - NLM (nih.gov)*
Kocuria flava Strain HO-9041	GCA_001482365.1	*Kocuria flava genome assembly ASM148236v1 - NCBI - NLM (nih.gov)*
